# A variable-trust threshold-based approach for DDOS attack mitigation in software defined networks

**DOI:** 10.1371/journal.pone.0273681

**Published:** 2022-08-29

**Authors:** Fatty M. Salem, Hoda Youssef, Ihab Ali, Ayman Haggag

**Affiliations:** 1 Department of Electronics and Communications Engineering, Faculty of Engineering, Helwan University, Cairo, Egypt; 2 Department of Electronics Technology, Faculty of Technology and Education, Helwan University, Cairo, Egypt; University College of Engineering Tindivanam, INDIA

## Abstract

Software-defined networks offer a new approach that attracts the attention of most academic and industrial circles due to the features it contains. However, some loopholes make such modern networks vulnerable to many types of attacks. Among the most important types of these attacks is the Distributed Denial of Service (DDoS) attack, which in turn affects the network’s performance and delays many real user requests. As one of the main features of SDN is the centralization of all the control plane in the SDN controller, it becomes a central point of attack that may compromise the whole network. Hence, in our proposed approach, we aim to mitigate the DDoS attack that maybe launched to compromise the SDN controller, flood the control plane and cripple the entire network. Many DDoS mitigation scheme have been proposed, however, determining the threshold between legitimate requests and malicious requests is still a challenging task. Our proposed approach relies on a two-phases algorithm that assigns a variable trust value for every user. This trust value is compared with schemes relying on a threshold value that changes dynamically and assists in detecting the DDoS attack. The first phase of our two-phases algorithm is Header fields extraction, and the second phase is calculating the trust value based on header fields information. Our proposed approach shows better performance than related detection schemes in terms of accuracy, detection rate, and false-positive rate. Where the accuracy of the system reaches up to 98.83% which is high compared to other traditional methods.

## 1. Introduction

Software-Defined Networking (SDN) is a recent paradigm in network management, where the network administrator can abstractly manage the network without knowing the details of the network. In general, the networks defined by the software consist of two levels: The Fuzzy Logic level [1] and Flow monitoring level [2]. The control plane is the central unit responsible for choosing the path for forwarding data in the network. It takes into account the receiver address and ensures the successful transmission of data through several data units distributed in the network called the data plane. The data plane in turn communicates with the requested user.

SDN is an emerging, dynamic, easy-to-manage, cost-effective, and adaptable architecture that separates network control and forwarding functions. Retargeting allows dynamic tuning of network traffic flow to meet changing needs. In the approach proposed in [3], SDN allows network administrators to quickly configure, manage, secure, and optimize network resources via dynamic and automated SDN software and provides comprehensive network visibility. SDN architecture enables or improves network-related security applications because it has a central view of the network and its ability to reprogram the data plane at any time. There is a lot of research already underway in security applications built on the SDN controller.

Detection and reduction of DDoS attack is an essential and important prerequisite for better performance of SDN networks. According to previous studies, the DDoS attacks are commonly carried out by **malicious agents against network systems**. Centralization of network control on a controller in the SDN architecture becomes a vulnerability exploited by DDoS attacks [4].

The authors in [5] suggested that the OpenFlow protocol is to be used to coordinate communication between the control plane and the data plane. Finally, authors in [6] proposed a security management solution for SDN using standard IPsec management framework.

The operation of SDN networks focuses on three basic tasks:

1. Centralized control and separate the control plane from the data plane.
2. More programmable and more flexible than traditional networks.
3. Reducing costs and dealing with networks easily.

The proposal in [7] identified several security attacks that SDN faces such as: data leakage, data modification, malicious/mixed applications, and denial of service attacks (DoS). This research paper focuses on the denial-of-service attack because of its great importance and its ability to threaten the network security and the penetration of software-defined network.

The network can be crippled by sending a large volume of data traffic to the network, making it impossible for another user in the network to receive services from network [8]. This leads to overloading the terminal network (such as switches, routers), or at least reduces the legitimate productivity of the network. The attacks can be driven by a variety of reasons; it could be for economic or political reasons. OpenFlow as a leading SDN protocol can used to help mitigate DDoS attacks. We can take advantage of separating the control plane and data plane to tackle DDoS problems.

The rest of the paper is organized as follows: Related work is overviewed in Section 2. The proposed approach is presented in Section 3. The simulation environment is given in Section 4. The results and performance are evaluated in Section 5. Finally, the paper is concluded in Section 6.

## 2. Related work

Despite the great openness and preference in using SDN on a large scale, it faces some challenges, the most important of which is the security problem. There are some researches have been carried out to solve network problems, including the Denial of Service (DoS) and Distributed Denial of Service (DDoS) attack because of their negative impact on the network and its performance.

From these researches is Avant-Guard [9] which is generally based on two main components: connection migration and actuating triggers. However, Avant-Guard is not effective in protecting the controller from DoS attacks using real IPs or through the proxy since Avant-Guard is limited to preventing TCP-based DoS attacks. Another weakness of Avant-Guard is its implementation on switches. All switches need to be Avant-Guard.

The scheme in [10] has been proposed to reduce denial of service attack; this approach simply cannot detect attackers who can change all field headers at once. This may be the case for an attacker who controls a large robot network. The mitigation can also lead to a relatively large set of table entries and performance degradation.

Also, one of the proposed solutions to confront the denial-of-service attack is Flowranger [11], which contributes to addressing the denial-of-service attack for the controller, which mainly depends on three main elements: trust management, queuing management, and scheduling requests. We find in this approach that it is not effective enough to deal with the denial-of-service attack because the root causes of this attack are not known, except that low priority requests are processed quickly, which may cause damage to some real users from dropping some packets or crashing due to the accumulation of requests.

The proposed scheme in [12] is based on monitor periods and threshold counters. Despite its effectiveness, it is linked to a specific time in which it can only detect the attack. S-Guard [13] offers a lightweight mechanism to avoid address spoofing, but it takes up bandwidth which affects the data plane significantly and network performance. Also, FloodShield [14] is one of the suggestions that helps in treating the DoS attack, and which also faces obstacles in verifying the source address where the attacker can impose his control on the original address of the packets and use them poorly.

SDN Gradient Descent [15] DoS attack prediction using gradient descent algorithm depends on the learning rate and according whether its high or low, this algorithm deals according to its value. On the other hand, a hybrid SDN-based approach [16] which is based on combining neural networks K-nearest neighbor (KNN) algorithm to reduce the false-positive rate.

In DoS defender [17], the DDoS defender application detects the DDoS attack by monitoring the number of flows in the OpenFlow switch. Once the volume of flows exceeds the predefined threshold the controller considers it a DDoS attack and inserts a flow rule to drop packets.

The authors in [18] have proposed a scheme to helps reduce DoS attacks, but it faces some challenges in complex storage and an increase in the number of hops, which leads to bandwidth consumption and cost increases. The authors in [19] proposed an effective platform for detecting DDoS attack, but it is also traditional and limited. The error rate is not negligible as it is affected by some real users. It is also associated with a specific threshold, which limits the flexibility to detect and identify the error more precisely and takes more time to detect DoS attack.

The authors in [20] proposed a lightweight using Self Organizing Maps (SOM) with high rate of detection and low rate of false alarm; however, the scheme didn’t allow of the communication between different detectors from different network domains.

The scheme in [21] uses machine learning to reduce the DDoS attack, specifically the low-rate DDoS attack, through the two phases of flow-based detection and mitigation. The authors in [22] introduced an approach to reduce DDoS attacks by using machine-learning-based algorithms, where the proposed model was trained with a radial kernel function that takes advantage of new and advanced features obtained from traffic flow information and statistics.

Based on deep learning, a spatial attention and convolutional neural network has been proposed in [23] for image-based classification of 25 well-known malware families. Internet of Things, which is clearly growing, also suffers from malicious threats, and the SDN model also provides a system for protection by controlling IoT devices safely. [24] is one from the research that dealt with reducing the DDoS attack of the IoT system. Table 1 shows a summary of our literature review for SDN security methods.

**Table 1:** Summary of our literature review.

Authors/reference	General description	Advantages	Disadvantages
Avant-Guard [9]	Based on two components: connection migration and actuating triggers.	Reduces the amount of data-to-control-plane interactions and insert conditional flow rules that are only activated when a trigger condition is detected.	Not effective in protecting the controller from DoS attacks using real IPs or through the proxy.
Scheme in [10]	A tailored statistical detection approach as well as a lightweight countermeasure	Can detect and mitigate attacks against the data plane in a lightweight and dependable way.	Cannot detect attackers who can change all field headers at once. The mitigation can also lead to a relatively large set of table entries and performance degradation.
Flowranger [11]	Depends on three main elements: trust management, queuing management, and scheduling requests.	Can significantly enhance the request serving rate of regular users under DoS attacks against the controller	Not effective enough to deal with the denial-of-service attack
S-Guard [13]	Uses a feature vector to classify traffic flows and optimizing classification by feature ranking and selecting algorithms.	Offers a lightweight mechanism to avoid address spoofing.	Takes up bandwidth which affects the data plane significantly and network performance.
FloodShield [14]	Combines source address validation which filters forged packets directly in the data plane, and stateful packet supervision.	Provides effective protection for all three components of the SDN infrastructure–data plane, control channel and control plane.	Faces obstacles in verifying the source address where the attacker can impose his control on the original address of the packets and use them poorly.
Platform in [19]	A platform to efficiently detect and rapidly respond to the DDoS attack in VNs based on software-defined networking (SDN)	The detection scheme effectively reduces the time for starting attack detection and classification recognition and has a lower false alarm rate.	The error rate is not negligible as it is affected by some real users.
Scheme in [20]	Lightweight using Self Organizing Maps (SOM).	High rate of detection and low rate of false alarm.	Didn’t allow of the communication between different detectors from different network domains.

The contribution of this research can be summaries as follows: Most of the proposed methods targeted DoS attacks, however, a more sever type of attack is DDoS where that attack is launched from several sources and is more difficult to mitigate. Our proposed mitigation algorithm is specially designed to mitigate the more serious DDoS attacks. Also, our proposed method is a two-phases algorithm based on assigning a variable trust value for every user relying on a threshold value that changes dynamically and assists in detecting the DDoS attack. Our statistical method used results in higher accuracy, detection rate, and false-positive rate for DDoS attacks.

## 3. The proposed approach

In our proposed approach, we try to find an effective solution that contributes to reducing the DDoS attack. There are many different ways in which the controller makes appropriate decisions for dealing with packet traffic. The attacker is likely to push many packets that need to be handled by the controller, which requires increasing the number of flow rules in switching tables. This causes the accumulation of new flows and an overload on the controller, which causes a large number of packets to fall out of the controller buffer.

The goal of our work is to find a solution to minimize the impact of the DDoS attack on the network so that real and regular users can perform their tasks well without suffering from poor network performance. An equation was designed from which the threshold changes dynamically, namely:

$$\text{th}\left(\text{s}\right)=\text{th}\left(\text{s}\right)+\text{tv}\left(\text{s}\right)$$ (1)

$$\text{th}\left(\text{s}\right)=\text{th}\left(\text{s}\right)-\text{tv}\left(\text{s}\right)$$ (2)

Eq (1) consists of the threshold th(s) added to the user’s trust value tv(s) which is used in the normal state of the network and in which there are no signs indicating the presence of an attack. Eq (2) consists of the user’s trust value tv(s) subtracted from the threshold th(s) which is used in the event of any signs that indicates an attack.

**Fig 1:**
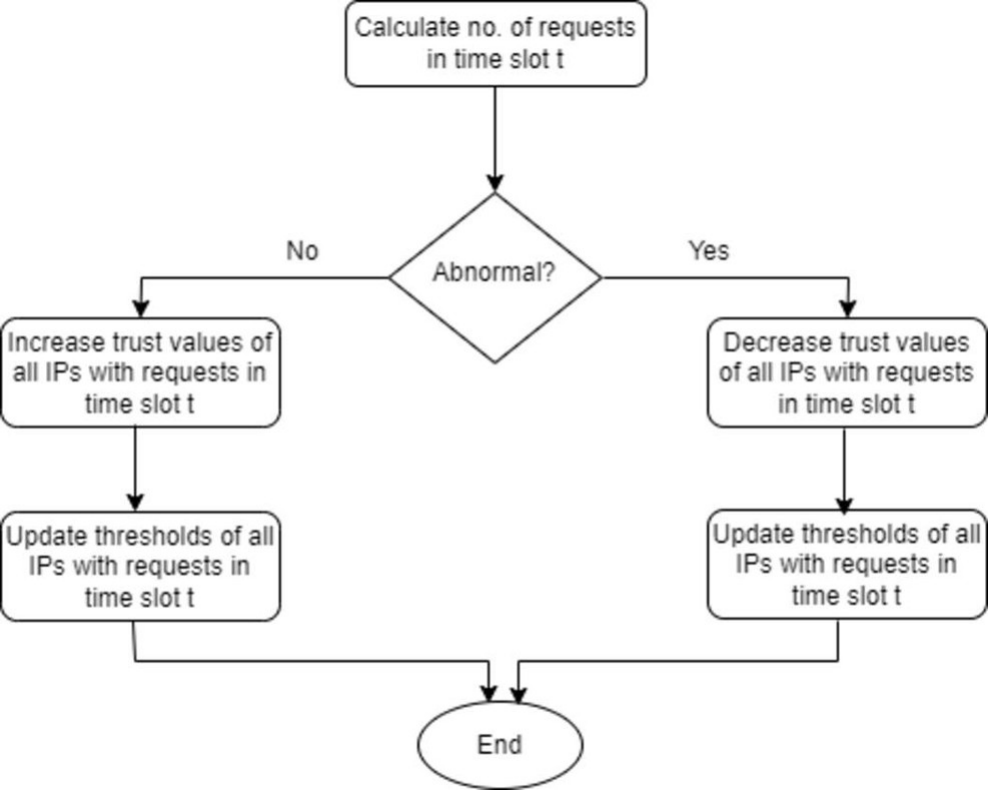
Steps of computing the threshold.

**Fig 2:**
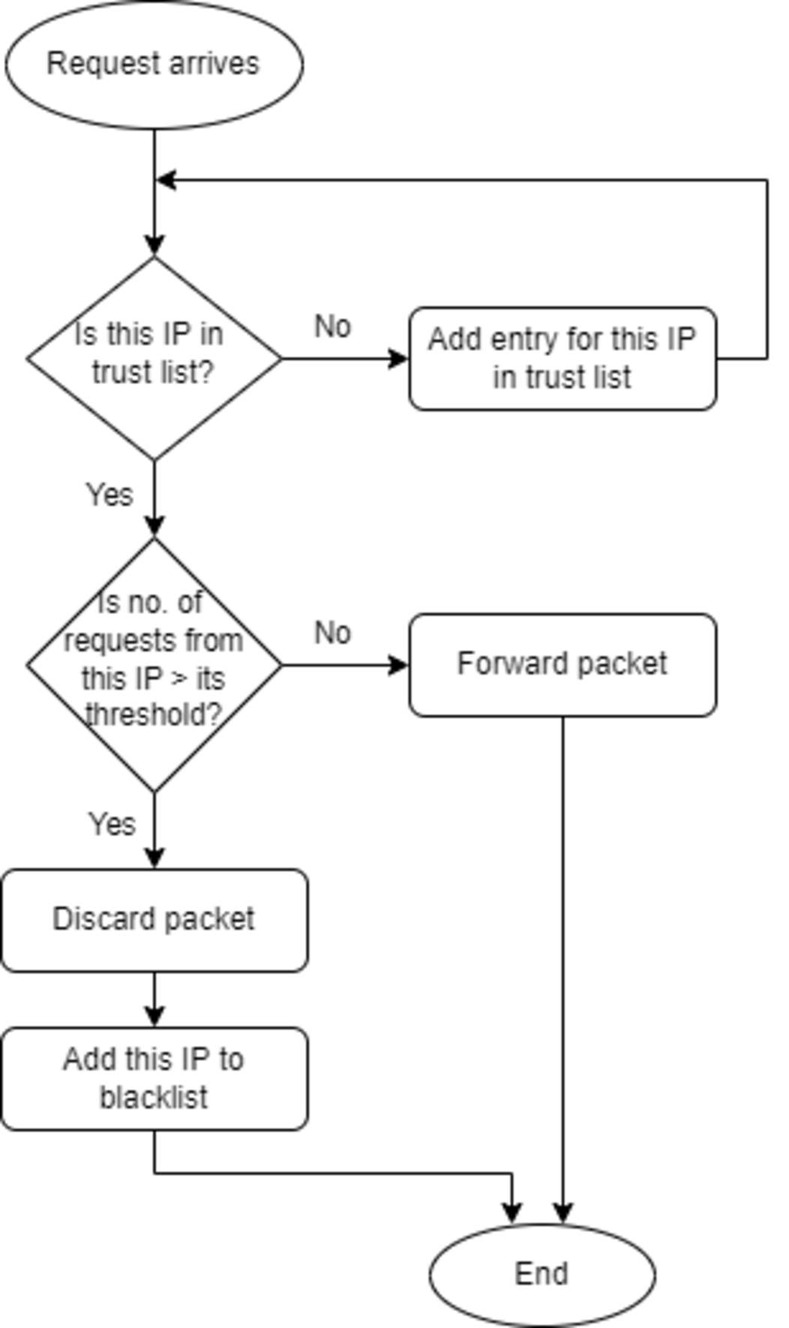
The proposed DDOS attack detection approach.

The proposed approach consists of two phases, namely: 1) Header fields extraction, 2) Calculating the trust value based on header fields information. Fig 1 shows the flow chart of the steps of computing the threshold and Fig 2 shows the flow chart of the proposed DDOS attack detection approach. Table 2 lists some notations used in the paper.

**Table 2:** List of notations.

Notation	Meaning
γ	The weight scale factor for header fields’ effect
L	Payload length header field
*X*	Payload length header field list
S	Source IP header field
$\vec{sl}$	Source IP header field list
d	Destination IP header field
$\vec{dl}$	Destination IP header field list
sp	Source port header field
$\vec{spl}$	Source port header field list
lth	Payload length header field threshold
sth	Source IP header field threshold
dth	Destination IP header field threshold
spth	Source port header field threshold
m_th_	The maximum threshold for user
N	Number of packets reaching network in this time slot
nm	Maximum number of packets reaching network in a normal state
$\vec{tv}$, tv_max,_ tv_min_	Trust value list, max, and min value
*S* *i*	SDN network user with index *i*
α	Forgetting factor, α ∈ (0,1)
θ*i*	Abnormal threshold for user *i*
T_min_	Threshold trust for malicious users
P_i_	Total request form user *S*_*i*_
N_q_	Number of priority buffer queues
L_max_	Total buffer length of N_q_ buffer queues
*Lj*	Length of the *j*^th^ buffer queue
*β*	Weight scale factor for buffer queues

### 3.1. Header field extraction

The proposed technique begins with finding out header fields of the SDN attack flow. The header fields are tracked to help in detecting DDoS attacks. The methodology uses a table of values as counters. The header fields are used as columns. During each specific time duration, the table values are compared against pre-defined thresholds. In case of a DDoS attack, the measured counted values of the header fields become larger than the pre-defined threshold values. On the other hand, when the network has normal traffic, not DDoS attack traffic, the values of the various header fields are smaller than threshold values.

**Fig 3:**
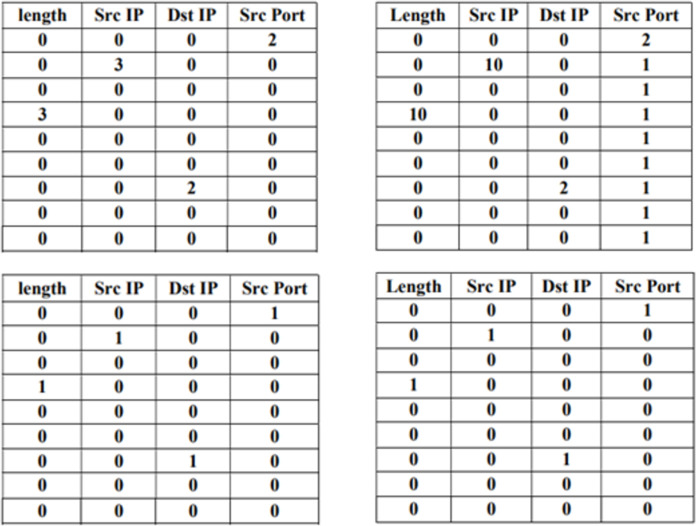
Simple example expressing a counter table growth overtime for ten consecutive packets with four header fields.

Fig 3 shows a simplified example showing the growth of a counter table over time for nine consecutive packets with four header fields: payload length, source IP, destination IP, and source port number where all are dynamic according to the values of headers in the incoming traffic. The columns represent the tracked header fields, while the rows are the number of times a value of the header field appears. The table cells are initialized to zero values. When a packet is received, the value of payload length is extracted. Hence, the table cell corresponding to this value in the payload length header field is incremented by one. The same operation is applied to the source IP, destination IP, and source port number header fields.

As a result of the proposed methodology, normal packets from legitimate users are served firstly with higher priority than traffic which is suspected to contain DDoS attack packets. Also, users who continuously send DDoS attack packets are blocked efficiently with high accuracy.

### 3.2. Calculating the trust value based on header fields information

Our main target through this paper is to protect legitimate users from the negative effects of attacking the SDN controller with a DDoS attack. To achieve our goal, the technique makes the controller serve packets through a user prioritization operation based on the attacking likelihood of the source with the aiding of taking into account tracking header field values and comparing them to the corresponding threshold. We utilize the information given from header fields of packets to calculate user’s trust value more efficiently. Algorithm 1 and algorithm 2 represent the operation of calculating trust management taking the information of tracking header fields into account.

### Algorithm 1. Function update trust according to headers

1.  Input: packet
2. Output: header_factor
3.  countl = 0
4.  counts = 0
5.  countd = 0
6.  countsp = 0
7.  count = 0
8. header_factor = 0
9. γ = 0.1
10.  extract header fields from packet
11.  if l not in ll then
12.  x (l) = 0
13.  else
14.  x (l) = x (l) + 1
15.  end if
16.  if s not in sl then
17.  sl (s) = 0
18.   else
19.  sl (s) = sl (s) + 1
20.  end if
21.  if d not in dl then
22.  dl (d) = 0
23.  else
24.  dl (d) = dl (d) + 1
25.  end if
26.  if sp not in spl then
27.  spl (sp) = 0
28.  else
29.  spl (sp) = spl (sp) + 1
30.  end if
31.  if (x (l) < lth) then
32.   countl + 1
33.  else
34.   countl − 1
35.  end if
36.  if (sl(s) < sth) then
37.   counts + 1
38.  else
39.   counts − 1
40.  end if
41.  if (dl(d) < dth) then
42.   countd + 1
43.  else
44.   countd − 1
45.  end if
46.  if (spl(sp) < spth) then
47.   countsp + 1
48.  else
49.   countsp − 1
50.  end if
51.  header_factor = header_factor + 0.25 * countl * γ
52.  header_factor = header_factor + 0.25 * counts * γ
53.  header_factor = header_factor + 0.25 * countd * γ
54.  header_factor = header_factor + 0.25 * countsp * γ

The preceding algorithm is a function that returns a factor that measures the effects of header fields’ values on computing the user’s trust value. The implementation of trust management is shown in algorithm 2.

### Algorithm 2. Trust management based on header fields’ information

1: In each time slot t
2: for each new packet p arriving at controller do
3: if the sender s is not in trust list tv then
4:  Add an entry for sender s in tv
5:  tv(s) = 1
6:  th(s) = tv(s) × 2
7: else
8:  if P_i_ < = th(s) then
     header_factor = update_trust_according_headers (packet)
9:   If n < = nm
10:    tv(s) = α × tv(s) + 1
11:    tv(s) = α × tv(s) + 0.01 × header_factor
12: th(s) = th(s) + tv(s)
13: th(s) = min(th(s), mth)
14: else
15: tv(s) = α × tv(s) - 1
16:    tv(s) = α × tv(s) + 0.01 × header_factor
17:    tv (s) = max(tv(s), 0)
18: th(s) = th(s)–tv(s)
19: th(s) = min(th(s), mth)
20:  end if
21: else
      discard packet
22: end if
    end if
23: if tv(s) < T_min_, then
24:  add the sender s as an attacker to the blacklist
25: end if
26: end for
27: In the end of each time slot t
28: for Each sender s in trust list do
29: if sender s does not appear in time slot t then
30:  tv(s) = α × tv(s)
31: end if
32: Update th(s) based on the total requests from the sender in time slot t
33: end for

If the source is well known, the algorithm updates the trust value and threshold. When the source sends the packet in a normal state, this gives an indication of being a legitimate source, hence the trust value and threshold must increase taking into account the effect of values of header fields as in Eqs (3), (4) and (5).


$$\text{tv}\left(\text{s}\right)=\alpha \times \text{tv}\left(\text{s}\right)+1$$ (3)

$$\text{tv}\left(\text{s}\right)=\alpha \times \text{tv}\left(\text{s}\right)+0.01\times \text{header}\_\text{factor}$$ (4)

$$\text{th}\left(\text{s}\right)=\text{th}\left(\text{s}\right)+\text{tv}\left(\text{s}\right)$$ (5)

The next step is to check if the source’s trust value is less than the minimum threshold of a legitimate source to block the source if this happens. When the SDN controller is suffering from a DDoS attack, the algorithm decreases the trust value and threshold taking the header field values as expressed in Eqs (6), (4) and (7).


$$\text{tv}\left(\text{s}\right)=\alpha \times \text{tv}\left(\text{s}\right)-1$$ (6)

$$\text{th}\left(\text{s}\right)=\text{th}\left(\text{s}\right)-\text{tv}\left(\text{s}\right)$$ (7)

Any source that does not send a packet during the time duration increases its trust value according to Eq (8).


$$\text{tv}\left(\text{s}\right)=\alpha \times \text{tv}\left(\text{s}\right)$$ (8)

In the algorithm shown, which aims to update and track the user’s trust values periodically in SDN networks, which depends on a dynamic change and trust value for each user, each according to its use, we maintain a list of regular users while also maintain the trust values for each of them that are updated in the case of use. As in the case of using the network in the normal state, the trust values for the regular users in the network are increased, but in the event that the network is exposed to any attack, the trust value decreases, as the length of the trust list depends on regular users, and the user is considered as an attacker and his request is immediately dropped and blacklisted.

## 4. Simulation environment

The experiments were carried out on a PC running Ubuntu 18.04.2 LTS OS. The system specifications are as follows:

CPU: Intel^@^ Core i7 CPU E7500 @ 3.4GHZ x 2
Memory: 7.9 GB

SDN simulation requires several controllers such as Pox [25], Ryu, and OPenDaylight. In our case, the best choice is the Pox controller. Pox controller is implemented using python. For SDN researchers, Pox controller is a common alternative. It is fast, lightweight, and can be tailored to suit a particular use. Pox controller is the improved version of their previous NOX controller [26].

### 4.1. Network emulator

Mininet [27], an emulator platform using OpenFlow protocol, uses lightweight virtualization to run a set of end-hosts, switches, routers, and links on a single Linux kernel. Mininet Modules function as real components of the network. The emulator has many tools to check potential bandwidth, node and deep node connectivity, and flow speed. Mininet is used by developers, students, and researchers due to its simple network connectivity using CLI and API, customization, and sharing features, as well as real hardware development features. Mininet is commonly used for quick developing of a simple network, supporting custom topologies and packet forwarding. Mininet is capable of running real programs on Linux, computers, servers, virtual machines, sharing and replicating resources, open-source and active development. In contrast to these advantages, Mininet also has several disadvantages like impairment of moving enormous quantities of data into a single system, unavailability of supporting arbitrary OpenFlow controllers, supporting only one platform (Linux kernel), sharing host file system and PID space, and absence of virtual time notion.

**Fig 4:**
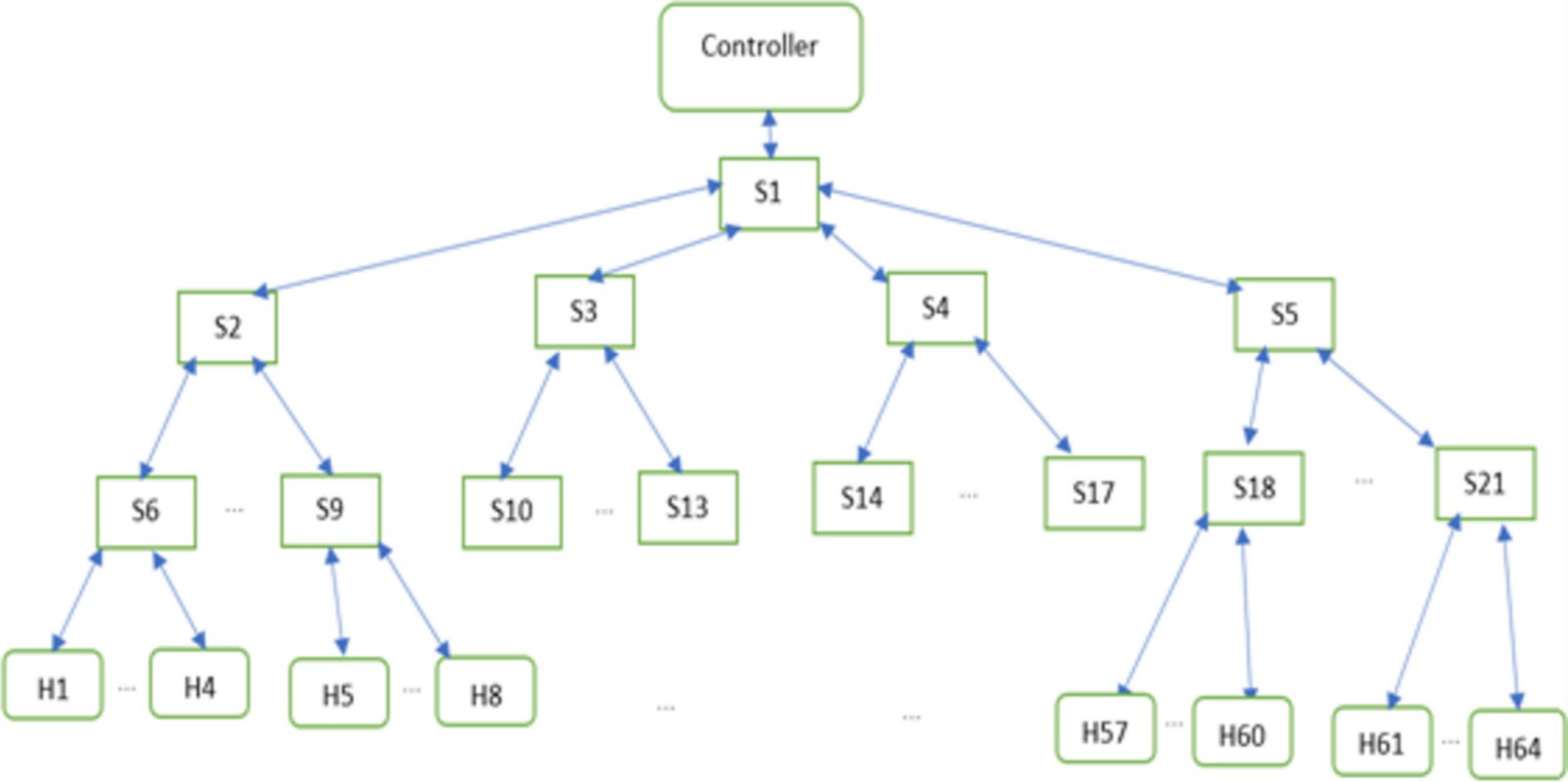
The network topology.

Using Mininet, a tree-type network with depth 21 switches, and 64 hosts was created as shown in Fig 4. For forwarding elements, OpenVswitch (OVS) [28] was used. Mininet simulation tool runs on a Linux machine operating under Ubuntu OS.

### 4.2. Traffic generation

Scapy [29] is a computer-network packet manipulation tool originally written by Philippe Biondi in Python. It can forge or decode packets, send them over the wire, catch them and match requests and answers. It can also perform activities such as scanning, tracerouting, searching, checking systems, attacking, and exploring networks. In native raw sockets, Scapy provides a Python interface close to that in which Wireshark provides a view and capture GUI. Scapy is also supporting packet injection, custom packet formats, and scripting which makes it better than other similar tools. Although it is just a command-line tool, it can still communicate with a variety of other programs to provide visualization, including Wireshark and GnuPlot to provide graphs, charts, etc [30].

### 4.3. Abstracted simulation

**Fig 5:**
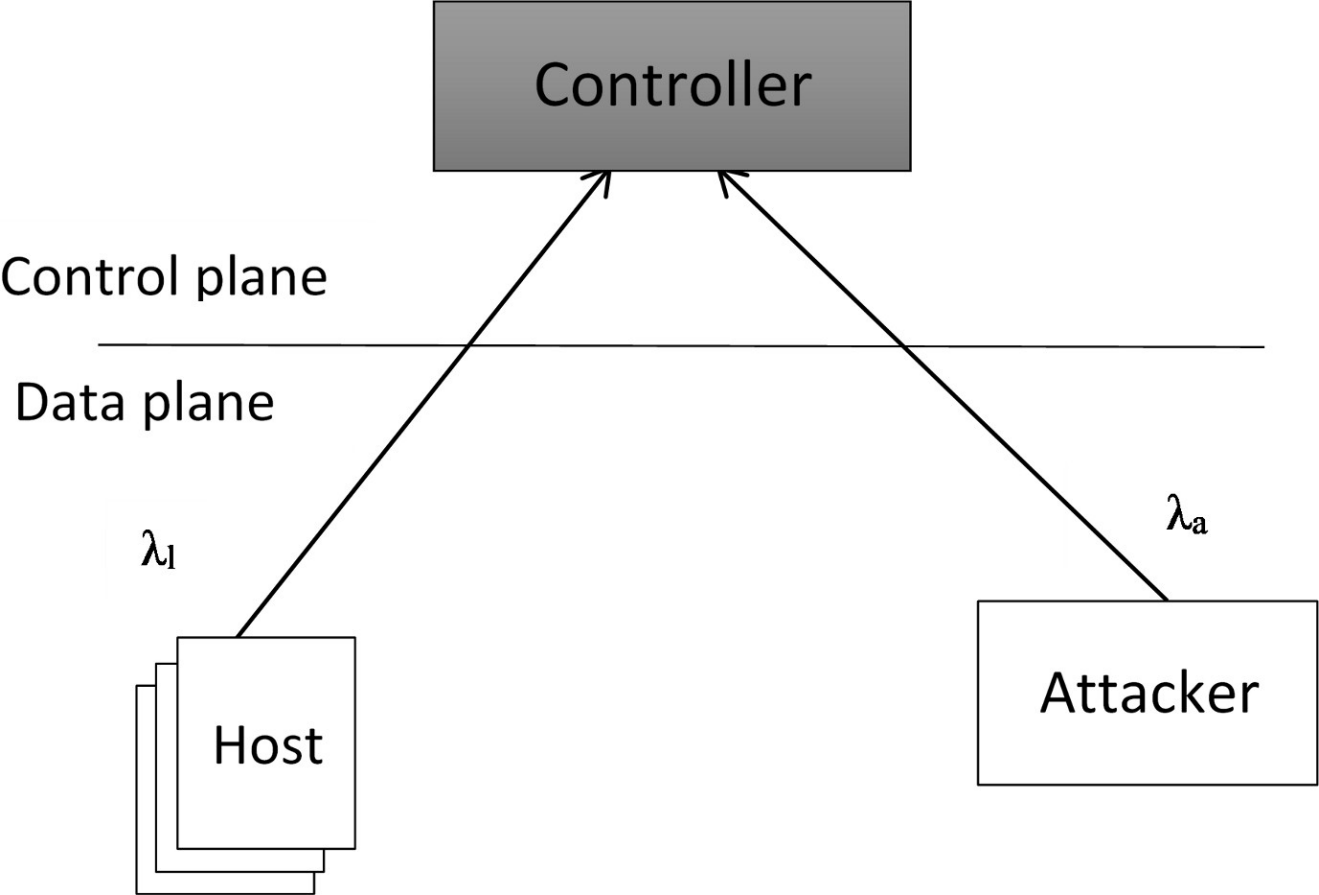
Abstracted simulation of the detection system.

To test the detection method, we designed a simulation based on Mininet [27]. One aspect of our simulation is that we did not simulate on the level of the data plane but also on the level of the control plane. The abstracted view is shown in Fig 5. When a new connection is initiated, i.e., with the first packet, a host in the simulated system triggers a new PacketIn. Both packets that belong to the same flow will ultimately be managed in the actual system in hardware (i.e., the data plane) and are not simulated. This significantly decreases the number of packets simulated, thus maintaining all of the attack’s important results.

In our simulation, we used negative exponentially distributed arrivals for valid users with an estimated mean interval time T_l_, corresponding to an arrival rate λ_l_ = 1 / T_l_. The arrival rate of the intruder is determined. The window size t_W_, i.e., the time between two consecutive runs of the detection algorithm, the detection threshold θ_m_, and the user threshold θ_u_ are other significant parameters. These parameters are expressed in Table 3.

**Table 3:** Parameters of simulation.

Notation	Meaning
λ_l_	Normal Traffic Arrival Rate
λ_a_	Attack Arrival Rate
t_W_	Window size
H	Number of Hosts
θ_m_	Max value system threshold
θ_u_	Max value user threshold

## 5. Results and performance evaluation

In this section, we demonstrate the evaluation methodology and show the results and performance of our proposed approach compared with other related approaches.

### 5.1. Evaluation methodology

Our detection mechanism approach marks the device state as "under attack" if the table limit reaches a certain threshold. The threshold should be low enough to detect all attacks, but if no attacks are attempted, it should not produce a warning. As usual, while undetected attacks are false negatives, we call these false alarms that are produced in the absence of an attack false positives. Empirically, the threshold could be set by simply trying different thresholds and measuring the accuracy of detection with the minimum rate of false-negative and false-positive alarms. Our system also has another type of threshold which is left as a variable adaptive threshold that depends on a trust value. Each host that sends packets of data over the network has its trust value. Consequently, each user has a private threshold calculated from the value of the user’s trust.

We simulated our technique with a network of legitimate H = 100 hosts, and 5 attacking devices. We utilized 10 seconds as a time slot between each checking of the existence of a DDoS attack. We use α = 0.9, defined as the forgetting factor in the trust value updating in the trust management system. The default number of queues in the priority buffers is *Nq* = 10 and the priority factor in request scheduling increase with a ratio of *β* = 1.5, while the trust increasing factor due to the value of a header field increase with a ratio of γ = 0.1. We set the minimum threshold for the user, which in case the user’s trust value becomes smaller than it, the user is considered as an attacker. The maximum number of packets for any user to send is assumed to be 24. The maximum number of packets that may reach the controller in the normal state in a one-time slot is set to 50 packets. Lmax is assigned 20 and the threshold of a header is 12.

We generate normal traffic with the inter-arrival time between every two successive packets of 0.1 sec and average arrival rate *λl* = 10 packets/sec. The attack traffic is generated with the small inter-arrival time between every two successive packets which is 0.01 sec and a high average arrival rate λa = 100 packets/sec. We set the maximum number of packets that can be received by the controller in the normal state as 50 packets. Otherwise, the controller is considered in the under-attack state.

True Positive (TP) is the number of attack states that are identified correctly, while False Negative (FN) is the number of attack states that are identified as normal. True Negative (TN) is the number of normal states correctly identified, while False Positive (FP) is the number of normal states identified as attacks.

Detection accuracy: it is the percentage between the number of truly described and labelled packets and the whole number of packets in the sample [31], as illustrated in Eq (9).


$$\text{Detection}\;\text{accuracy}=\frac{\text{TP}+\text{TN}}{\text{TP}+\text{TN}+\text{FP}+\text{FN}}$$ (9)

False-positive rate: It is the percentage between the number of legitimate packets incorrectly classified as attack and the whole number of legitimate packets [31]. Eq (10) formulates the false positive rate.


$$\text{False}-\text{positive}\;\text{rate}=\frac{\text{FP}}{\text{FP}+\text{TN}}$$ (10)

Detection rate: it is the percentage between the correctly considered and labelled threat packets and the whole number of threat packets [31], as defined in the next equation.


$$\text{Detection}\;\text{rate}=\frac{\text{TP}}{\text{TP}+\text{FN}}$$ (11)

### 5.2. Results and comparisons

**Fig 6:**
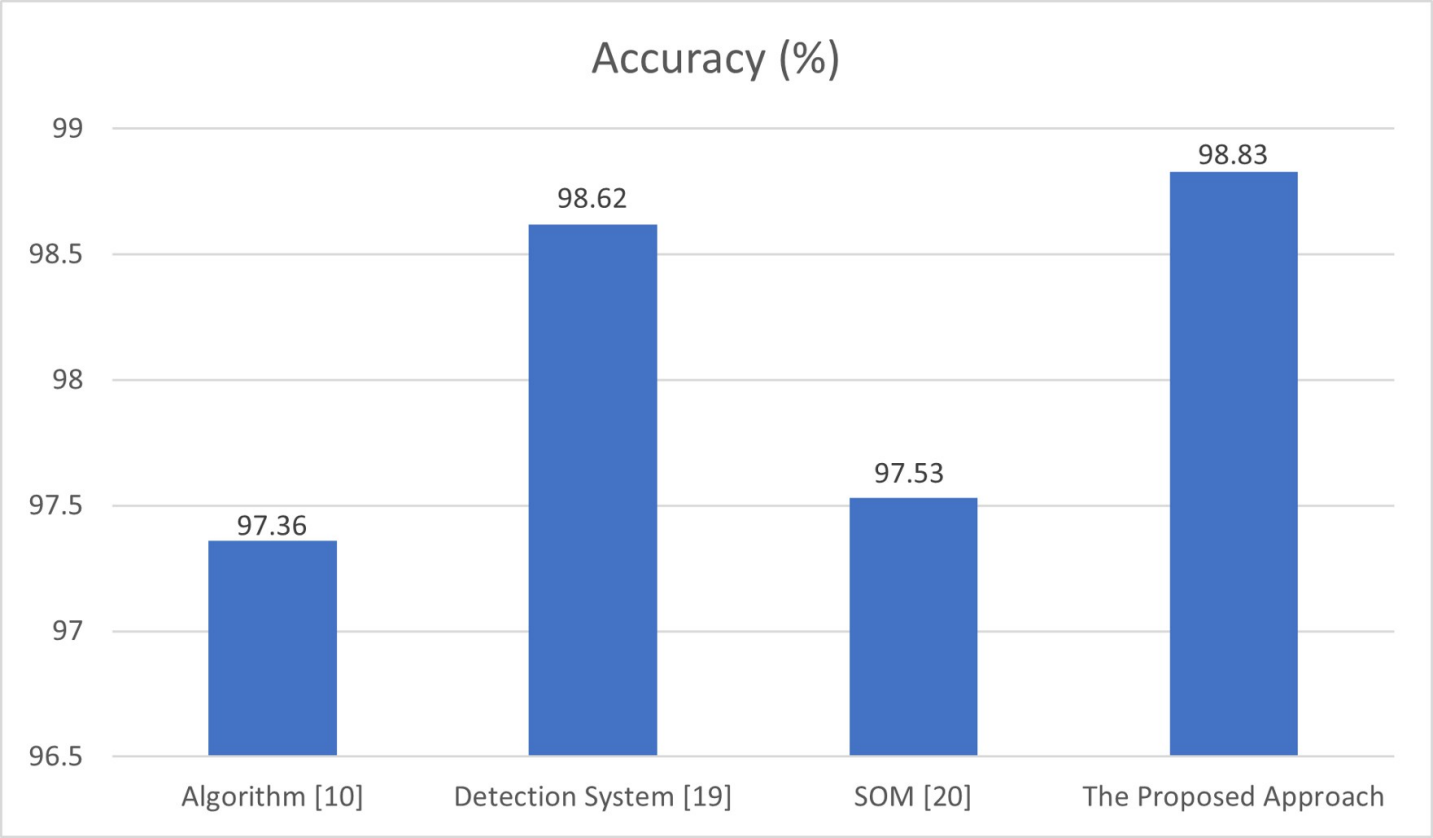
Comparison of accuracy of algorithm [10], detection system [19], detection method [20], and the proposed approach.

The behavior of our approach is compared against related techniques that detect DDoS attacks in SDN. It is compared with the algorithm presented in [10], the detection system implemented in [19], and the SOM-based DDoS flooding attack detection [20]. The proposed approach provides better detection accuracy than these implemented algorithms. The accuracy is evaluated for the proposed approach under the increasing rate of data traffic. Fig 6 depicts that the proposed approach provides high detection accuracy of 98.83%, while the algorithm in [10] provides a detection accuracy of 97.36, the detection system in [19] provides a detection accuracy of 98.62%, and the detection method in [20] provides 97.53%. These results demonstrate that the proposed approach provides the best performance in accuracy compared to other related studies.

**Fig 7:**
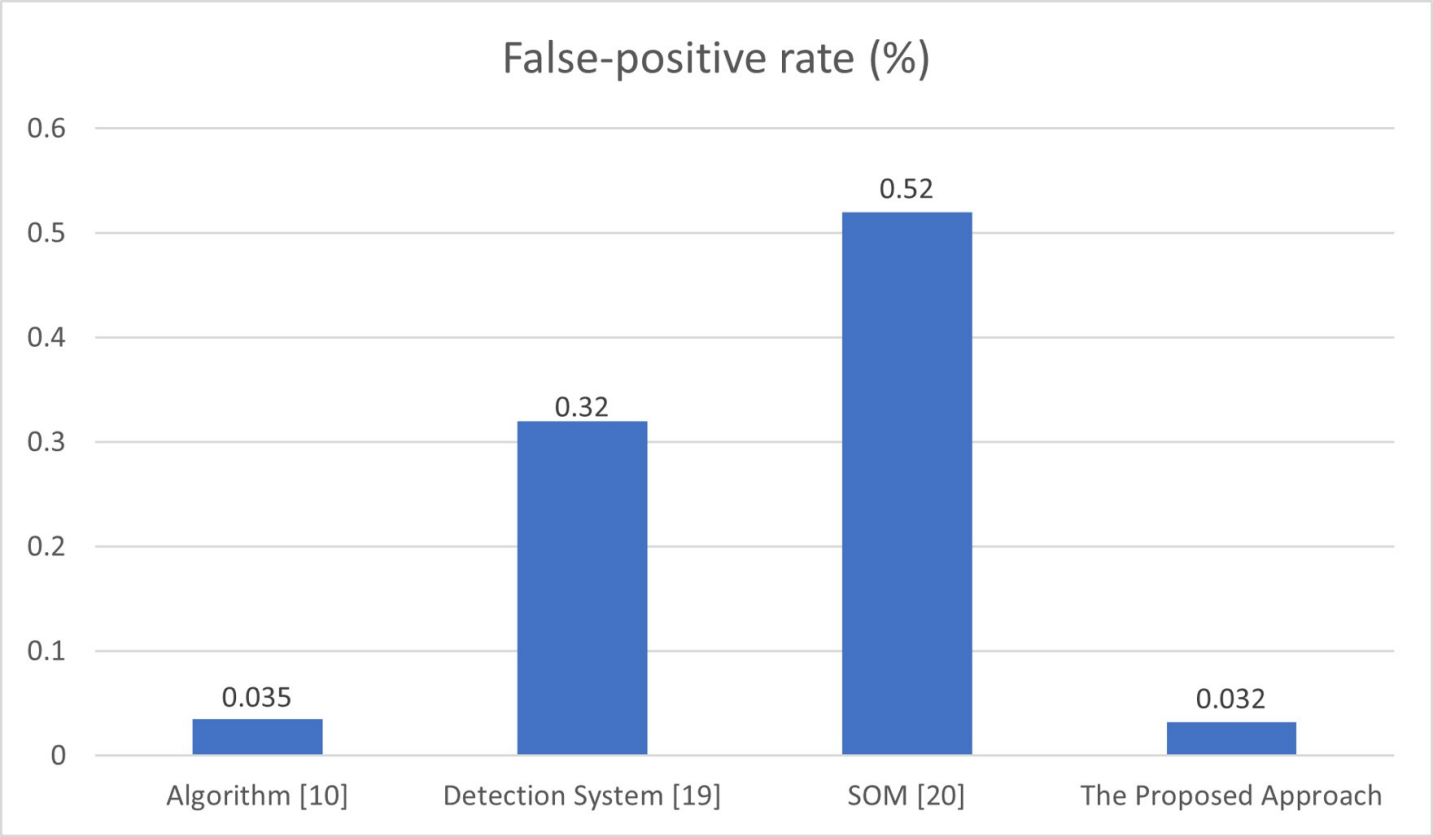
Comparison of false-positive rate of algorithm [10], detection system [19], detection method [20], and the proposed approach.

Another performance metric is used to measure the performance of introduced approach and other related methods, which is the false-positive rate. Fig 7 expresses the behavior of our proposed approach against the algorithm with constant threshold which is presented in [10], the detection system implemented in [19], and the SOM-based DDoS flooding attack detection [20] from the perspective of the false positive rate.

As described in Fig 7, the proposed approach provides a false positive rate measures 0.032%; however, the algorithm introduced in [10] gives 0.035%, the detection system in [19] provides 0.32%, and the detection method in [20] offers 0.52%. Hence, the proposed approach provides the least false positive rate compared to other implemented methods. The minimal false positive rate of the proposed approach is mainly facilitated due to the utilization of a variable threshold in the process of distinguishing malicious flow from the normal flow in SDN networks.

**Fig 8:**
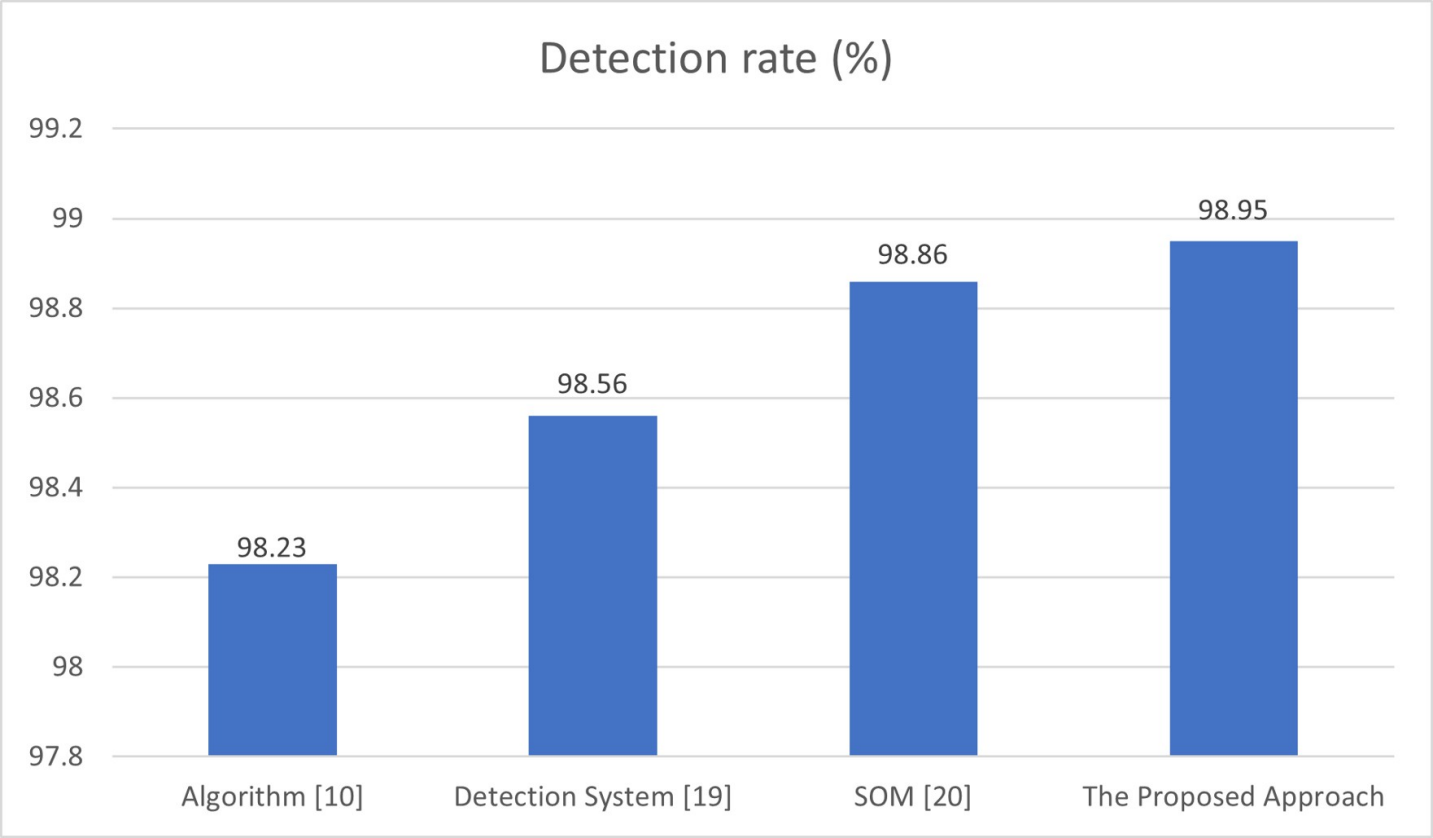
Comparison of detection rate of algorithm [10], detection system [19], detection method [20], and the proposed approach.

The detection rate is also another effective performance measure, which is tracked to compare the performance of the proposed approach in this paper and other algorithm. Fig 8 shows the results of the comparison of detection rate of algorithm in [10], detection system in [19], the SOM-based DDoS flooding attack detection [20], and the proposed approach. Fig 8 depicts that the proposed approach has a detection rate of 98.95%, while algorithm [10] has a detection rate of 98.23, the detection system in [19] has a detection rate of 98.56%, and the detection method in [20] has a detection rate of 98.86%. Hence, the proposed approach provides a better detection rate than the schemes in [10, 19, 20]. The improvement in the performance is because the proposed approach uses a variable threshold instead of using a fixed threshold.

**Fig 9:**
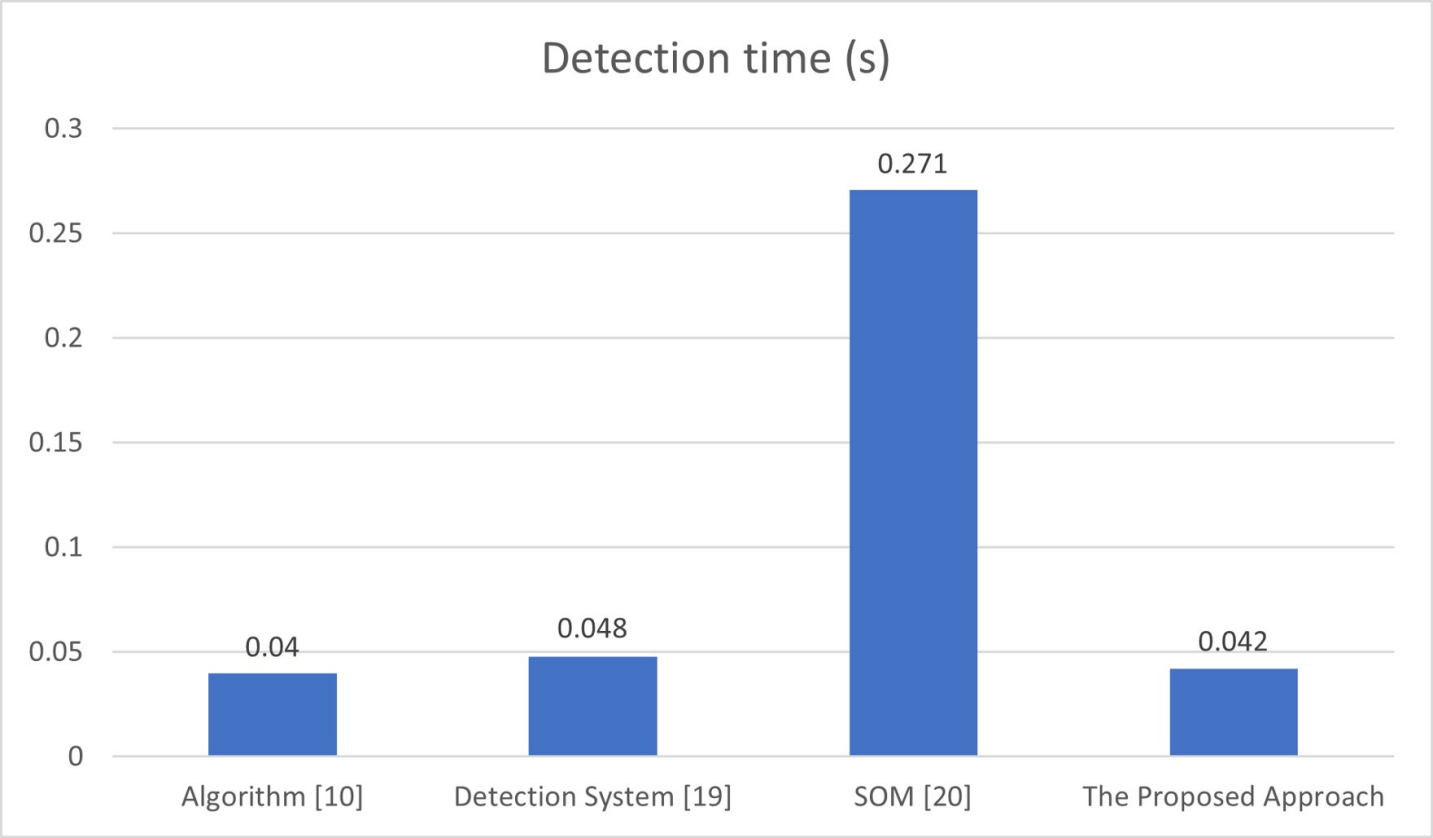
Comparison of detection time of algorithm [10], detection system [19], detection method [20], and the proposed approach.

In addition to all previous performance metrics, there is an additional powerful measure, which is the detection or classification time. The detection time is the average delay needed by the technique or system to decide that there is an attack or the network’s state is normal. As shown in Fig 9, a comparison of the detection time in the proposed scheme, the algorithm with constant threshold which is presented in [10], the detection system implemented in [19], and the SOM-based DDoS flooding attack detection [20] is provided.

Fig 9 depicts that the proposed approach has needs 0.042s to complete the detection process, while algorithm [10] needs 0.04s, the detection system in [19] needs 0.048s, and the detection method in [20] needs 0.271s. Hence, the proposed approach has comparable time to the schemes in [10, 19, 20].

In summary, the proposed variable-trust threshold-based approach can effectively cope with DDoS attacks. In addition, the experiment results all demonstrate that the detection process of the proposed approach is faster than other schemes relying on constant threshold.

## 6. Conclusion

In our proposed approach, we present two-phases detection approach using two algorithms for the two phases that help in the early detection of a DDoS attack by enhancing the trust value for real users and lowering the trust value for the malicious users. Our proposed approach is based on the trust value that changes by changing the threshold dynamically below its maximum limit. If the number of requests exceeds a certain limit, the trust value for this user is reduced and considered as an attacker. This allows us to detect attacks and drop them immediately which can be achieved by examining the header fields table. Depending on the number of requests, if it remains below a certain number of requests over a period of time, the user is considered to be a realistic user. On the other hand, if the number of requests exceeds the maximum limit, the user is considered as an attacker and his packets are dropped. Our assessment shows that the attack can be detected by the algorithms in our two-phases detection approach, dealt with explicitly, and the network performance is improved with a higher accuracy and detection rate, lower false-positive rate, and reasonable short detection time. In future work, we will consider the detection of distributed denial of service attack in case that the attacker can change field headers at once as we didn’t consider this case in the proposed approach.
